# The differentiated and conserved roles of Swi5‐Sfr1 in homologous recombination

**DOI:** 10.1002/1873-3468.12656

**Published:** 2017-05-08

**Authors:** Bilge Argunhan, Yasuto Murayama, Hiroshi Iwasaki

**Affiliations:** ^1^ Institute of Innovative Research Tokyo Institute of Technology Japan

**Keywords:** Dmc1, DNA repair, genome stability, homologous recombination, Rad51, Swi5‐Sfr1

## Abstract

Homologous recombination (HR) is the process whereby two DNA molecules that share high sequence similarity are able to recombine to generate hybrid DNA molecules. Throughout evolution, the ability of HR to identify highly similar DNA sequences has been adopted for numerous biological phenomena including DNA repair, meiosis, telomere maintenance, ribosomal DNA amplification and immunological diversity. Although Rad51 and Dmc1 are the key proteins that promote HR in mitotic and meiotic cells, respectively, accessory proteins that allow Rad51 and Dmc1 to effectively fulfil their functions have been identified in all examined model systems. In this Review, we discuss the roles of the highly conserved Swi5‐Sfr1 accessory complex in yeast, mice and humans, and explore similarities and differences between these species.

## Abbreviations

**co‐IP**, coimmunoprecipitation/coimmunoprecipitate

**DSBs**, double‐strand breaks

**dsDNA**, double‐stranded DNA

**ES**, embryonic stem

**HJ**, Holliday junction

**HR**, homologous recombination

**NHEJ**, nonhomologous end joining

**ssDNA**, single‐stranded DNA

Even in the absence of radiation and mutagenic chemicals, DNA incurs damage due to the by‐products of healthy cellular metabolism. Moreover, the process of replicating DNA before each cell division has a propensity to generate double‐strand breaks (DSBs) in DNA, resulting in broken chromosomes. Such damage can lead to genomic instability, a hallmark of cancer. Two major DNA repair pathways protect our genomes from DSBs: homologous recombination (HR) and nonhomologous end joining (NHEJ) 1, 2.

HR‐mediated DSB repair is dependent on the presence of an intact stretch of DNA that shares high sequence similarity with the broken DNA molecule (i.e. is homologous). In contrast, NHEJ does not involve a homologous partner and the broken ends are ligated together. NHEJ is therefore thought to be inherently more mutagenic than HR. Although first observed during the meiotic cell cycle, HR has since been shown to be involved in a variety of biological processes other than DSB repair, including the generation of immunological diversity as well as numerous aspects of chromosomal biology such as telomere length regulation and ribosomal DNA maintenance 1.

**Figure 1:**
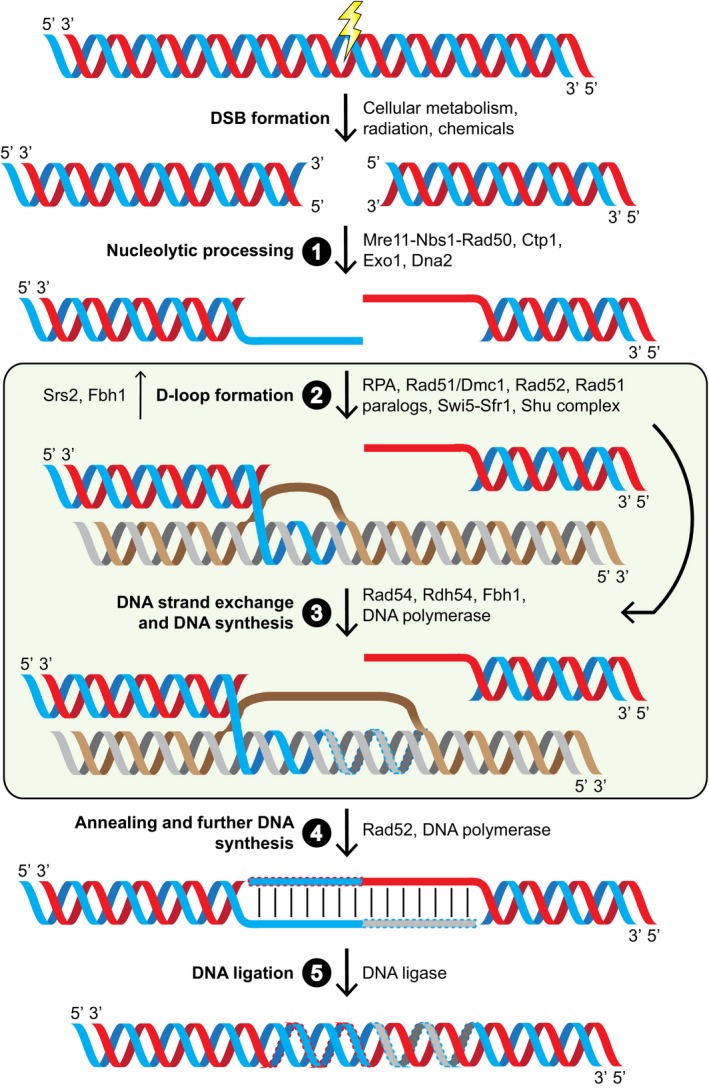
Homologous recombination is a major DSB repair pathway. (1) A DSB forms and the dsDNA undergoes nucleolytic processing to reveal ssDNA. Loading of ssDNA‐binding protein RPA removes secondary structures which would otherwise impede HR. (2) With assistance from recombination accessory proteins, the eukaryotic recombinases Rad51/Dmc1 displace RPA and form a right‐handed helical filament around the ssDNA. This nucleoprotein filament, also known as the presynaptic filament, identifies homologous dsDNA and invades into it, displacing the noncomplementary strand to form a displacement loop (D‐loop). (3) The recombinases catalyse strand exchange as the invading 3′ end is elongated through DNA synthesis. The curved arrow from (2) denotes that at least some of those proteins also participate in promoting DNA strand exchange. (4) This invading strand is ejected, and having been extended using the intact duplex as a template, is able to anneal to the ssDNA on the other side of the DSB. This opposing ssDNA now uses the extended strand as a template for further DNA synthesis, and following the reformation of hydrogen bonds between the complementary strands, dsDNA containing single‐strand nicks is generated. (5) These nicks are repaired by a DNA ligase, resulting in the completion of HR‐mediated DSB repair. This model is a depiction of synthesis‐dependent strand annealing, a HR pathway that does not yield crossover products.

The central proteins in HR are the Rad51 and Dmc1 recombinases, which are the eukaryotic homologues of the bacterial recombinase RecA. These RecA family recombinases are defined by their ability to directly promote strand exchange in a homology‐dependent manner and differ from classical site‐specific recombinases. Although Rad51 is required for both mitotic and meiotic HR 3, 4, 5, Dmc1 is produced in a meiosis‐specific manner and thus only contributes to meiotic HR 6, 7. In a broad sense, recombinases facilitate two molecular processes that lie at the heart of HR: identification of and subsequent invasion into intact homologous double‐stranded DNA (dsDNA), and DNA strand exchange. However, recombinases cannot perform these tasks alone. Studies have led to the identification of proteins other than recombinases that are required for HR in all examined model organisms (see ref. 8 for an in‐depth review). Of particular relevance to the regulation of Rad51 activity are the Rad52 family of proteins, Rad51 paralogues, the Swi5‐Sfr1 complex and the Shu complex (recently reviewed in ref. 9). Genetic and biochemical evidence has suggested that these different groups of recombination accessory proteins are highly conserved and have nonoverlapping roles in regulating HR (Table 1) 10. Moreover, the requirement for each group seems to vary depending on the species examined. Why several groups of accessory proteins are required for HR and to varying extents in different organisms is not well understood, primarily due to a lack of insight into the precise molecular mechanism of HR (Fig. 1).

**Table 1:** Common recombination accessory proteins among eukaryotes. A mediator is defined as a factor that allows Rad51 to overcome the inhibitory effect of RPA by utilising RPA‐coated ssDNA for strand exchange. An activator is a factor that can stabilise the filament and/or stimulate strand exchange through another, uncharacterised means (e.g. stimulation of ATP hydrolysis by Rad51)

Function^a^	*Schizosaccharomyces pombe*	*Saccharomyces cerevisiae*	*Mus musculus*/*Homo sapiens*
Mediator	Rad52	Rad52	RAD52/BRCA2
Mediator, activator	Rad55‐Rad57	Rad55‐Rad57	RAD51C‐XRCC3, RAD51B‐RAD51C‐XRCC3
Mediator (activator?)	Rlp1‐Sws1‐Rdl1^b^	Shu1‐Shu2‐Psy3‐Csm2	SWS1‐RAD51D‐XRCC2
Activator	Swi5‐Sfr1	Sae3‐Mei5^c^	SWI5‐SFR1

a Function is denoted with regard to Rad51. Note that BRCA2 and Swi5‐Sfr1 also stimulate Dmc1 activity *in vitro*.

b Rlp1 = Shu1 = XRCC2, Sws1 = Shu2 = SWS1, Rdl1 = Psy3 = RAD51D.

c Sae3‐Mei5 is a meiosis‐specific mediator and activator of Dmc1.

Since the last review on Swi5‐Sfr1 11, numerous advances have been made in the field, including extensive biochemical and structural characterisation of fission yeast (*Schizosaccharomyces pombe*) Swi5‐Sfr1 and the budding yeast (*Saccharomyces cerevisiae*) homologue Mei5‐Sae3. Furthermore, the discovery of the Swi5‐Sfr1 recombination accessory complex in mammals has provided support for the notion that proteins involved in fundamental processes such as DNA repair are highly conserved throughout evolution. In this review, we will focus on the roles of Swi5‐Sfr1 in HR. We will first describe the identification and *in vivo* characterisation of Swi5‐Sfr1 in yeast, before turning our attention to the biochemical characteristics of this protein complex. A mechanistic model for the role of Swi5‐Sfr1 in HR will be proposed, followed by a description of how Swi5‐Sfr1 was identified and characterised in mammals, and how it compares with its yeast counterpart. Finally, we will discuss the implications of these findings in a broader context and consider the key questions that remain to be answered.

## Fission yeast Swi5‐Sfr1: insights from genetics

Early genetic studies in both *S. cerevisiae* and *S. pombe* led to the characterisation of numerous genes that are required for functional mitotic and meiotic HR. These genes encode recombination accessory proteins such as Rad52 5, 12, 13, 14 and the Rad51 paralogue complex Rad55‐Rad57 15, 16, 17, 18, 19. Although these genes were originally isolated in a screen for mutations that confer sensitivity to radiation 20, the discovery of Swi5 in *S. pombe* was linked to mating‐type switching, a phenomenon that occurs in some homothallic fungi and allows the conversion of cell type through an HR‐dependent DNA rearrangement (reviewed in ref. 21, 22). The *swi5* ^*+*^ gene was originally identified as a gene required for mating‐type switching in *S. pombe* 23 and later studies demonstrated that *swi5* ^*+*^ was one of the few *swi* genes also required for resistance to radiation‐induced DNA damage 24. In a seminal study, the mating‐type switching protein Swi2 was shown to interact with Swi5 and Rad51 *via* its C‐terminal half 25. However, unlike the *swi5* ^*−*^ and *rad51* ^*−*^ mutants, the *swi2* ^*−*^ mutant did not show any sensitivity to DNA damage, suggesting that the function of Swi2 within the Swi5‐Swi2‐Rad51 ensemble is specific for mating‐type switching. Although this explained why mating‐type switching is defective in the *swi5* ^*−*^ mutant, it failed to explain the DNA damage sensitivity seen in the *swi5* ^*−*^ mutant. The authors hypothesised that Swi5 might form a complex with another Rad51‐interacting protein to promote HR in a more general context, at locations other than the mating‐type locus. Consistent with this possibility, an unannotated gene with homology to the C terminus of Swi2 was identified. Furthermore, the protein product of this gene contained a putative Rad51‐binding region and was shown to interact with Swi5 and Rad51. Importantly, the deletion mutant was proficient for mating‐type switching but as sensitive to radiation‐induced DNA damage as the *swi5* ^*−*^ mutant. This protein was named Sfr1 for Swi five‐dependent recombination repair protein 1.

Subsequently, several studies reinforced the notion that Swi5‐Sfr1 is important for HR in *S. pombe*. First, it was demonstrated that the Rad51‐dependent repair of meiotic DSBs, which are highly induced through the programmed activation of the topoisomerase‐like enzyme Spo11, requires Swi5 26, 27. Second, Swi5 and Sfr1 foci were shown to colocalise with Rad51 in response to radiation‐induced DNA damage 28. Furthermore, the formation of Rad51 foci, which likely represent Rad51 nucleoprotein filaments, was impaired in the absence of Swi5, suggesting a role for Swi5‐Sfr1 in stabilising Rad51 filaments. Third, the *sfr1* ^*−*^ mutant showed sensitivity to a plethora of chemicals that induce DNA damage, much like the *rad55* ^*−*^ */rad57* ^*−*^ mutants 29. However, for all examined parameters, the phenotype of the *swi5* ^*−*^ */sfr1* ^*−*^ mutant was not as severe as the *rad51* ^*−*^ mutant.

These genetic data suggested that, in the absence of Swi5/Sfr1, Rad51 activity is reduced but not abolished. In support of this notion, the DNA damage sensitivity of the *swi5* ^*−*^ *rad57* ^*−*^ double mutant was indistinguishable from the *rad51* ^*−*^ mutant 25, and the formation of Rad51 foci was more defective in the *swi5* ^*−*^ *rad57* ^*−*^ double mutant than either single mutant 28. Moreover, overproduction of Rad51 almost completely suppressed the UV sensitivity of the *swi5* ^*−*^ and *rad57* ^*−*^ single mutants, but only partial suppression was observed in the *swi5* ^*−*^ *rad57* ^*−*^ double mutant 18, 25, 28, suggesting that a high concentration of Rad51 can effectively bypass the requirement for Swi5‐Sfr1 or Rad55‐Rad57, but not both. It was proposed that two independent pathways for Rad51‐dependent HR exist in *S. pombe*: the Rad55‐Rad57 pathway and the Swi5‐Sfr1 pathway 28. Implicit in this model was the idea that Swi5‐Sfr1 and Rad55‐Rad57 differentially regulate Rad51, with each protein complex performing a distinct function to achieve full Rad51 activity. Consistently, experiments involving the induction of a site‐specific DSB showed that, while Rad51‐dependent crossover formation was reduced in the *sfr1* ^*−*^ mutant, it was completely abolished in the *rad57* ^*−*^ mutant, strongly suggesting that Rad57, but not Sfr1, plays an essential role in promoting crossover formation 28. Moreover, overproduction of Rad57 did not suppress the defects seen in the *swi5* ^*−*^ or *sfr1* ^*−*^ mutants, and overproduction of Swi5 or Sfr1 was unable to suppress the defects associated with the *rad57* ^*−*^ mutant 11. These findings provided the first major hints that the role of Swi5‐Sfr1 in promoting HR was distinct from previously characterised recombination accessory factors such as Rad55‐Rad57.

This hypothesis was substantiated by subsequent genetic analyses involving Rrp1 (TTF2 in *Homo sapiens*) and Rrp2 (HLTF in *H. sapiens*), which are both homologues of *S. cerevisiae* Uls1 with predicted helicase and ATPase activities 30. Although the *rrp1* ^*−*^ or *rrp2* ^*−*^ single mutants did not show sensitivity to DNA damage, the *rad57* ^*−*^ *rrp1* ^*−*^ and *rad57* ^*−*^ *rrp2* ^*−*^ double mutants were more sensitive to DNA damage than the *rad57* ^*−*^ single mutant. In fact, these double mutants were almost as sensitive to DNA damage as the *rad57* ^*−*^ *sfr1* ^*−*^ double mutant (and the *rad51* ^*−*^ single mutant). In contrast, the DNA damage sensitivity of the *sfr1* ^*−*^ *rrp1* ^*−*^ and *sfr1* ^*−*^ *rrp2* ^*−*^ double mutants resembled the *sfr1* ^*−*^ single mutant. Importantly, the *rad51* ^*−*^ *rrp1* ^*−*^ and *rad51* ^*−*^ *rrp2* ^*−*^ double mutants showed the same DNA damage sensitivity as the *rad51* ^*−*^ mutant, indicating that *rad51* ^*−*^ is epistatic to *rrp1* ^*−*^ and *rrp2* ^*−*^. Taken together, these data suggested that Rrp1 and Rrp2 have a role specifically in the Swi5‐Sfr1 pathway of HR. Consistent with this model, yeast‐two hybrid data indicated that Rrp1 and Rrp2 interact not just with each other, but also with Swi5 31. Given the similarity between the *rrp1* ^*−*^ and *rrp2* ^*−*^ mutant phenotypes, it is highly likely that the two proteins form a functional complex *in vivo* to regulate Swi5‐Sfr1‐specific HR. This specialised regulation of the Swi5‐Sfr1 HR pathway by Rrp1‐Rrp2 provides further evidence for the idea that recombination accessory factors differentially influence the molecular process of HR. The role of Rrp1‐Rrp2 in HR remains to be determined, but the most recent model proposed by Dziadkowiec *et al*. 31 places this complex in the Swi5‐Sfr1 pathway of HR, where it functions alongside the Srs2 helicase to promote gene conversion events.

Following the discovery of Swi5‐Sfr1 in *S. pombe*, a homologous protein complex called Sae3‐Mei5 was identified in *S. cerevisiae*. *SAE3* was identified as a gene that is required for very similar processes as *DMC1* during meiosis, with both mutants undergoing prophase I arrest and accumulating DSBs 32. However, unlike its *S. pombe* orthologue *swi5* ^*+*^, *SAE3* is only expressed during meiosis and the *sae3* mutant does not show any sensitivity to DNA‐damaging agents 32, strongly suggesting that the role of Sae3 is restricted to meiosis. Likewise, Mei5 was produced in a meiosis‐specific manner and the *mei5* mutant showed defects only in meiosis 33. Subsequently, *dmc1* was shown to be epistatic to both *sae3* and *mei5* for numerous meiotic phenomena including spore formation, spore viability and DSB repair 34. Moreover, it was demonstrated by immunofluorescence microscopy that Dmc1, Sae3 and Mei5 foci largely colocalised on meiotic chromosomes 34, suggesting that Sae3 and Mei5 localise to sites of ongoing Dmc1‐dependent DSB repair. Although the formation of Dmc1 foci was dependent on Sae3 and Mei5, the formation of Rad51 foci was independent of both factors, strongly suggesting that Sae3 and Mei5 regulate Dmc1 but not Rad51 during meiosis. Several subsequent findings provided strong support for this hypothesis. Through the use of a physical assay that directly monitors DNA, it was demonstrated that the formation of recombinant DNA molecules during meiosis was greatly reduced in the *mei5* mutant 35. The existence of a Sae3‐Mei5 complex was also verified through *in vivo* coimmunoprecipitation/coimmunoprecipitate (co‐IP) experiments 35. Finally, and most importantly, *in vivo* co‐IP assays demonstrated conclusively that Mei5 forms a complex with Dmc1 during meiosis; yeast two‐hybrid analysis indicated that this interaction required the N terminus of Mei5 35. Taken together, these findings provided compelling evidence that Sae3‐Mei5 function together as a meiosis‐specific recombination accessory complex that specifically promotes the activity of Dmc1, but not Rad51.

## Recombinase mediators and the inhibitory effect of RPA

An *in vitro* three‐strand exchange assay was used to demonstrate that Rad51 from *S. cerevisiae* (*Sc*Rad51) facilitates pairing of circular single‐stranded DNA (ssDNA) with homologous linear dsDNA and subsequent strand exchange in an ATP‐dependent manner 36. Interestingly, the inclusion of ssDNA‐binding protein RPA in the three‐strand exchange reaction stimulated DNA strand‐exchange, indicating that RPA plays a stimulatory role in HR 36, 37. However, further experiments suggested that addition of RPA to ssDNA before or simultaneously with Rad51 leads to reduced Rad51 activity due to competition between Rad51 and RPA for ssDNA binding 37. This observation highlighted a conundrum in the field of HR. If RPA is required for efficient HR, but it also competes with Rad51 for ssDNA binding, how does Rad51 displace ssDNA‐bound RPA to facilitate DSB repair? The answer to this question came through the characterisation of a group of recombination accessory proteins known as mediators. Here, we will reserve the term mediator for recombination accessory factors that are able to efficiently overcome the inhibitory effect of RPA on strand exchange *in vitro* 38. The first eukaryotic mediator to be extensively studied was *Sc*Rad52. Several groups independently demonstrated that *Sc*Rad52 can overcome the inhibitory effect of RPA in the three‐strand exchange reaction 39, 40, 41. Although RAD52 from *H. sapiens* (*Hs*RAD52) could stimulate RAD51‐mediated DNA pairing *in vitro*, these experiments did not involve RPA 42. Importantly, more recent experiments demonstrated that the breast cancer‐associated gene product BRCA2, which does not have a homologue in *S. pombe* or *S. cerevisiae*, stimulates *Hs*RAD51 *in vitro* by overcoming the inhibitory effect of RPA 43. Thus, BRCA2, but not *Hs*RAD52, serves as a canonical recombination mediator in humans 43. In contrast to *Hs*RAD52 and *Sc*Rad52, BRCA2 is unable to efficiently anneal RPA‐coated ssDNA. It is likely that BRCA2 and *Hs*RAD52 have assumed divergent roles in mammalian cells throughout the course of evolution, with BRCA2 taking on the role of mediator and *Hs*RAD52 taking on the role of annealer.

Following the characterisation of Rad52, other recombination accessory proteins were studied. The *S. cerevisiae* Rad51 paralogues Rad55 and Rad57 were purified as a complex and shown to stimulate strand exchange by overcoming the inhibitory effect of RPA 44. More recent experiments utilising budding yeast proteins demonstrated that the Shu complex (consisting of Shu1‐Shu2‐Csm2‐Psy3) acts synergistically with Rad52 and Rad55‐Rad57 to stimulate Rad51 loading and strand exchange *in vitro* 45. A distant relative of the Shu complex was identified in *S. pombe* (consisting of Rlp1‐Sws1‐Rdl1), although a homologue of the Csm2 subunit has not been discovered 46. In contrast to budding yeast, human RAD51 paralogues have not yet been shown to have a clear stimulatory effect on RAD51 *in vitro* 2. Even less is known about the biochemical properties of Rad51 paralogues and the Shu complex in *S. pombe*, as there are no reports detailing their purification.

## Biochemical characterisation of yeast Swi5‐Sfr1

Extensive *in vivo* evidence suggested that Swi5‐Sfr1 is also a recombination accessory factor that directly promotes Rad51 activity (discussed in Fission yeast Swi5‐Sfr1: insights from genetics). Conclusive evidence in support of this hypothesis came through the demonstration that Swi5‐Sfr1 can stimulate both Rad51‐mediated strand exchange and Dmc1‐mediated strand exchange *in vitro* 47. However, in the case of Rad51, this stimulatory effect was substantially reduced when ssDNA was preincubated with RPA, leading to suggestions that the mechanism whereby Swi5‐Sfr1 stimulates strand exchange is distinct from that of *Sc*Rad52 and *Sc*Rad55‐Rad57. To begin addressing this possibility, Rad52 from *S. pombe* (*Sp*Rad52) was purified and its effect on the strand exchange reaction was examined 48. Interestingly, the inhibitory effect of RPA was completely abolished when both Rad52 and Swi5‐Sfr1 were included in the reaction, suggesting that the two accessory factors synergistically stimulate the displacement of RPA by Rad51. This was shown more directly by challenging RPA‐saturated ssDNA with Rad51 in the presence of either/both Rad52 and Swi5‐Sfr1. Two subsequent observations clarified the distinct roles of Rad52 and Swi5‐Sfr1 in strand exchange. Firstly, Rad52, but not Swi5‐Sfr1, was able to interact simultaneously with Rad51 and ssDNA‐bound RPA. Secondly, Swi5‐Sfr1, but not Rad52, was shown to enhance the ssDNA‐dependent ATPase activity of Rad51 and protect ssDNA‐bound Rad51 against displacement by RPA in an ATP‐dependent fashion. Taken together, these findings lead to a model where Rad52 recruits Rad51 to ssDNA through a direct interaction with RPA before collaborating with Swi5‐Sfr1 to promote Rad51 filament formation by facilitating RPA displacement. Swi5‐Sfr1 then stabilises and activates the Rad51 filament in an ATP‐dependent manner. Consistently, experiments utilising flow linear dichroism have suggested that Swi5‐Sfr1 induces a conformational change in the Rad51 filament 49. In addition, Swi5‐Sfr1 was shown to oppose the disruption of Rad51 filaments by the F‐box DNA helicase Fbh1, further supporting the notion that Swi5‐Sfr1 stabilises Rad51 filaments 50.

This characterisation of Rad51‐mediated strand exchange was followed by an investigation into the roles of Swi5‐Sfr1 and Rad52 in Dmc1‐mediated strand exchange 51. In contrast to Rad51‐mediated strand exchange, Swi5‐Sfr1 was able to efficiently stimulate Dmc1‐mediated strand exchange even when ssDNA was preincubated with RPA, indicating that Swi5‐Sfr1 alone is able to negate the inhibitory effect of RPA on Dmc1. Likewise, Swi5‐Sfr1 alone was able to promote the efficient displacement of ssDNA‐bound RPA by Dmc1 and the stabilisation of ssDNA‐bound Dmc1 against RPA. These findings strongly suggest that Swi5‐Sfr1 alone fulfils the role of a canonical recombination mediator for Dmc1. Consistent with this notion, and in stark contrast to Rad51, these mediator activities of Swi5‐Sfr1 were largely negated by the presence of Rad52, indicating that Rad52 negatively regulates Dmc1‐mediated HR. It should be noted that neither Rad51 nor Dmc1 have a strict requirement for Swi5‐Sfr1 in the four‐strand exchange assay, which mimics the formation and branch migration of Holliday junctions (HJ) 52, 53, suggesting that Swi5‐Sfr1 does not play a substantial role in HR following second‐end capture.

What is the significance of Swi5‐Sfr1 during meiosis? Genetic observations have established that *Sp*Rad52 is not required for crossover formation during meiosis 54. Moreover, physical assays detecting recombinant DNA molecules indicated that Rad52 is dispensable for interhomologue HJ formation, whereas Swi5‐Sfr1 is essential; these dependencies are reversed for intersister HJ formation 55. Taken together, these *in vitro* and *in vivo* data lead to a model where Swi5‐Sfr1 takes on the role of a canonical mediator during meiosis in addition to its usual role in strand exchange activation to promote Dmc1‐dependent interhomologue recombination.

Results obtained from biochemical experiments with Sae3‐Mei5, the meiosis‐specific homologue of Swi5‐Sfr1 in *S. cerevisiae*, strongly supported the notion that this complex promotes the activity of Dmc1 only. Sae3‐Mei5 was shown to overcome the inhibitory effect of RPA on Dmc1 in the D‐loop assay, which measures strand invasion 56. Likewise, Sae3‐Mei5 facilitated Dmc1 filament formation in the presence of RPA 56. Subsequent analysis directly demonstrated that Sae3‐Mei5 interacts with Rad51 through the N‐terminal half of Mei5 but does not promote Rad51‐mediated strand exchange 57, thus confirming the notion that Sae3‐Mei5 only stimulates Dmc1 activity in budding yeast. Nonetheless, these data raised questions about the functional significance of the Rad51–Mei5 interaction. A potential explanation for this was provided by Cloud *et al*. 58, who proposed a noncatalytic role for Rad51 in collaborating with Sae3‐Mei5 to promote Dmc1 activity. Although these findings provide an attractive model for the role of Sae3‐Mei5 in *S. cerevisiae* meiosis, they also highlight the need to determine how Swi5‐Sfr1 differentially modulates Rad51 and Dmc1 activity during meiosis in *S. pombe*.

## Molecular model for Rad51 activation by Swi5‐Sfr1

Structural studies were instigated to gain further insight into the role of Swi5‐Sfr1 in HR. Attempts to crystallise the full length Swi5‐Sfr1 complex were unsuccessful, so Swi5 in complex with a 180 residue N‐terminal deletion mutant of Sfr1 (Sfr1C) was crystallised instead 59. This early analysis revealed that Swi5 and Swi5‐Sfr1C exist as tetramers and heterodimers in solution, respectively. Subsequent small‐angle X‐ray scattering experiments showed that the Swi5‐Sfr1 complex is nonspherical and has an elongated dogleg‐shaped structure 60. This has been corroborated by ion mobility mass spectrometry 61. Computer modelling of the Rad51 filament was combined with this structure of the Swi5‐Sfr1 complex, leading to suggestions that Swi5‐Sfr1 fits into the grooves of the Rad51 filament to maintain the elongated, active form of the filament 60. This model also indicated that it is Swi5 that enters the groove while Sfr1 plasters along the side of the filament, with the Sfr1 N terminus being most distal to the groove. Furthermore, the N‐terminal half of Sfr1 (Sfr1N) was shown to interact with DNA and co‐IP with Rad51, but it was unable to stimulate Rad51 activity *in vitro* 62. In contrast, the C‐terminal half of Sfr1 in complex with Swi5 stimulates Rad51 activity but does not co‐IP with Rad51, indicating that that any physical interaction between Swi5‐Sfr1C and Rad51 is weak or transient (Swi5 alone does not co‐IP with Rad51 47). Interestingly, Swi5‐Sfr1C did not inhibit Rad51 at high concentrations, as the full‐length complex does, suggesting that the stimulation conferred by Swi5‐Sfr1C is somewhat aberrant. Kuwabara *et al*. 62 resolved the crystal structure of Swi5‐Sfr1C and in doing so revealed that the molecule is sharply kinked. Taken together, these results point towards a model where the N‐terminal of Sfr1 anchors the Swi5‐Sfr1 complex to the Rad51 filament through direct interactions with Rad51. Subsequently, the C‐terminal of Sfr1, in complex with Swi5, enters the groove to maintain the filament in the active form. Structural data of Swi5‐Sfr1 homologues are required before we can begin to speculate on whether this mechanism of recombinase stimulation is evolutionary conserved.

**Figure 2:**
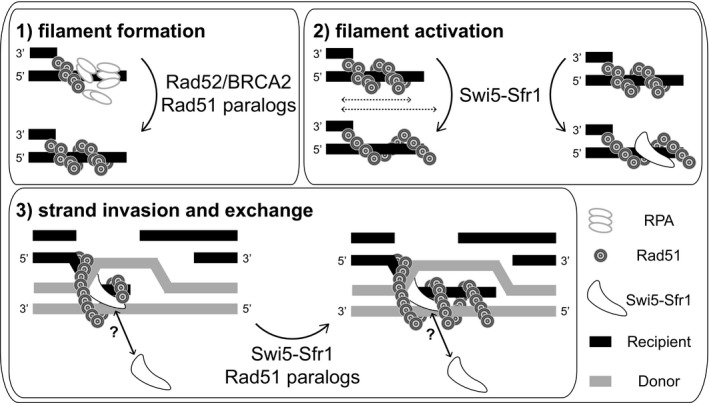
Stimulation of Rad51 activity by Swi5‐Sfr1. (1) Rad51 alone cannot displace RPA from ssDNA to form a filament. Mediators such as Rad52/BRCA2 and Rad51 paralogues facilitate the removal of RPA from ssDNA and subsequent formation of the Rad51 filament. This filament has relatively low strand exchange activity. (2) The Rad51 filament is restructured into a stabilised form, likely due to the insertion of Swi5‐Sfr1 into the grooves of the filament. This active filament is extended compared to the inactive filament. (3) Following strand invasion, Swi5‐Sfr1 stimulates ATP hydrolysis by Rad51 to enhance strand exchange, likely by making the filament more dynamic. Whether Swi5‐Sfr1 remains part of the groove or exerts an effect on strand exchange through a different mechanism is not known. Rad51 paralogues likely play a role here too.

How does Swi5‐Sfr1 promote Rad51 activity? As mentioned above, the presence of Swi5‐Sfr1 stabilises the Rad51 filament in an ATP‐dependent manner, preventing the displacement of Rad51 by RPA 48. Interestingly, a similar stabilisation of the Rad51 filament can be achieved without Swi5‐Sfr1 through the inclusion of the nonhydrolysable ATP analogue AMP‐PNP 48. However, Swi5‐Sfr1 can promote efficient DNA strand exchange of plasmid‐sized DNA, whereas AMP‐PMP cannot 48. These results not only indicate that a stable filament alone cannot catalyse strand exchange, but crucially, they suggest that the role of Swi5‐Sfr1 in stimulating Rad51 extends beyond filament stabilisation. ATP binding and hydrolysis by Rad51 is thought to be linked to association with and dissociation from ssDNA, respectively, leading to a model where dynamic changes in the filament must occur to facilitate efficient strand exchange 63. Based on these findings, we propose that Swi5‐Sfr1 stimulates Rad51 activity in two ways. First, by inserting into the grooves of the Rad51 filament, Swi5‐Sfr1 stabilises the filament and renders it resistant to displacement. We refer to this as the presynaptic role of Swi5‐Sfr1. Second, once the stabilised Rad51 filament invades into homologous duplex DNA, we speculate that Swi5‐Sfr1, through an unknown mechanism, enhances Rad51‐dependent strand exchange by stimulating ATP hydrolysis (Fig. 2). The elucidation of a postsynaptic role for Swi5‐Sfr1, which is the first recombination accessory factor to have such a role ascribed to it, is hugely important to understanding the molecular mechanism of HR.

## The discovery of SWI5‐SFR1 in mammals

Based on amino acid conservation, homologues of Swi5 and Sfr1 were identified in mice (*Mus musculus*) 64. Much like their yeast counterparts, SWI5 and SFR1 were shown to form a complex *in vivo* and *in vitro*, and both proteins localised to the nucleus. Furthermore, SWI5 protein could not be detected in *Sfr1* ^*−/−*^ mouse embryonic stem (ES) cells, and SFR1 levels were diminished in *Swi5* ^*−/−*^ cells, strongly suggesting that association of SWI5 with SFR1 enhances the stability of both proteins *in vivo*. Consistent with an evolutionarily conserved role for SWI5‐SFR1 in HR, *in vitro* co‐IP experiments demonstrated that SWI5, but not SFR1, interacts with RAD51, whereas the opposite is true in *S. pombe* 47. Contrary to expectations, the levels of *in vivo* HR, measured through the use of a fluorescence‐based assay, were not affected by the absence of either SWI5 or SFR1. Nonetheless, *Swi5* ^*−/−*^ and *Sfr1* ^*−/−*^ cells were more sensitive than wild‐type to chemicals that cause DNA strand breaks. In an attempt to reconcile these results, the BRC3 peptide, which is known to inhibit HR, was expressed in mouse ES cells. A 10‐fold reduction in HR was seen in wild‐type cells, but a 20‐fold reduction was seen in cells lacking SWI5 or SFR1. Taken together, these results suggest that SWI5‐SFR1 does promote HR in mice, but that its contribution to HR only becomes significant under conditions where HR is already compromised.

Extensive *in vitro* characterisation of mouse SWI5‐SFR1 followed its identification. In an oligonucleotide strand exchange assay, SWI5‐SFR1 was shown to stimulate RAD51 activity 65. SWI5‐SFR1 was also able to protect RAD51‐ssDNA complexes against exonucleolytic digestion, suggesting that it is able to stabilise RAD51 filaments 65. Importantly, these stimulatory effects were completely lost when only SWI5 or SFR1 was included in the reactions, suggesting that complex formation is essential for SWI5‐SFR1 function. Subsequent analyses revealed that SWI5‐SFR1 also stimulates the ssDNA‐dependent ATPase activity of RAD51, likely by enhancing the rate of ADP release 66. Furthermore, mutational analysis led to the identification of F83 and L85 in the SWI5 C terminus as residues that are important for the functional interactions with RAD51 67. Both of these residues are highly conserved, being identical in humans (F229 and L231) and highly similar in *S. pombe* (F79 and V81).

Shortly after the discovery of SWI5‐SFR1 in mice, a homologous complex was identified in *H. sapiens* 68. Although the homologous proteins were referred to as SWI5‐MEI5 throughout that article, we will maintain the mouse terminology for simplicity. At 235 amino acid residues, the human SWI5 protein is substantially larger than both the *S. pombe* and mouse homologues (85 and 89 residues, respectively). Interestingly, both subunits of human SWI5‐SFR1 are capable of binding to RAD51. Moreover, in contrast to what was seen with a fluorescence‐based HR assay in mouse cells, the absence of SWI5 or SFR1 in human cells led to a clear reduction in HR. Accordingly, these mutant cell lines displayed increased sensitivity to ionising radiation. Taken together, these data provide compelling evidence that the role of SWI5‐SFR1 as a recombination accessory factor has been conserved throughout evolution.

## Similarities and differences between yeasts and mammals

There are numerous noteworthy attributes of the Swi5‐Sfr1 complex that are conserved from yeast to mammals. In this section, we will discuss some of the more striking similarities and differences.

The nature of the Swi5–Sfr1 interaction is of interest. In *S. pombe*, the C‐terminal half of Sfr1 is required for the interaction with Swi5; since Swi5‐Sfr1C but not Sfr1N can stimulate Rad51, it is likely that Swi5‐Sfr1 complex formation is essential for its function 62. Consistent with this finding, the C‐terminal half of mouse SFR1 and the N‐terminal half of SWI5 are required for SWI5‐SFR1 complex formation 64. In humans, the C‐terminal half of SFR1 as well as the C‐terminal half of Swi5 is required for the interaction 68. Although this might seem to differ from *S. pombe* and mice, it is important to note that the N terminus of human SWI5 has an extension that does not exist in most other species. Regardless of this difference, in each case, the module required for the interaction contains a highly conserved coiled‐coil motif 68, suggesting that this feature lies at the heart of Swi5‐Sfr1 complex formation.

The interaction between Swi5‐Sfr1 and Rad51 is thought is to be essential for Swi5‐Sfr1 function. In *S. pombe*, it was shown that Swi5‐Sfr1, but not Swi5 alone, interacts with Rad51 47. Moreover, Sfr1N but not Swi5‐Sfr1C co‐IPs with Rad51, indicating that the Rad51 interaction motif lies in the N‐terminal half of Sfr1 62. In stark contrast, mutation of two C‐terminal residues within mouse SWI5 (F83A and L85A) was shown to abolish the functional interaction between SWI5‐SFR1 and RAD51 without affecting the SWI5–SFR1 interaction 67. Despite this, evidence from independent groups suggests that the SWI5‐SFR1 complex has higher affinity for RAD51 than either individual subunit 64, 65, consistent with observations in *S. pombe* 62. A unique feature of the human SWI5‐SFR1 complex is that both subunits can interact with RAD51, although the interaction between SFR1 and RAD51 is relatively weak 68.

In addition to these structural differences, there are numerous physiological differences. In *S. pombe*, Swi5 has Sfr1‐independent roles in mating‐type switching 25; there is currently no evidence to suggest that mammalian SWI5 functions in an SFR1‐independent manner. Furthermore, although the *swi5* ^*−*^ and *sfr1* ^*−*^ mutants in *S. pombe* are indistinguishable with regard to DNA damage sensitivity 25, 28, this is not the case in mammals. Although *swi5* ^*−/−*^ and *sfr1* ^*−/−*^ mouse cells showed similar sensitivity to ionising radiation, *sfr1* ^*−/−*^ cells were more sensitive to camptothecin and etoposide, which are type I and type II topoisomerase poisons, respectively 64. In contrast, human cells lacking SWI5 were more sensitive to ionising radiation than cells lacking SFR1, although this may simply reflect the fact that siRNA knockdown of SWI5 was more efficient than SFR1 68. The significance of these differences in the requirement for SWI5 and SFR1 in DNA repair is not currently understood.

In both *S. cerevisiae* and *S. pombe*, Swi5‐Sfr1 plays an integral role in meiotic HR; this is reflected in its ability to serve as a canonical mediator for Dmc1 *in vitro* 47, 51, 56. In contrast, mouse SWI5‐SFR1 did not stimulate DMC1 *in vitro* 65, but considering that a meiosis‐specific isoform of SWI5 has been reported in mouse testes 64, and SWI5‐SFR1 is expressed in the human germline 68, it is expected that mammalian SWI5‐SFR1 indeed plays some role in meiotic HR, at least through the regulation of RAD51, which was recently demonstrated to play an essential role in mouse meiosis 69. Further research is required to uncover the precise role of SWI5‐SFR1 in mammalian meiosis.

## Conclusions and perspectives

There is no doubt that Swi5‐Sfr1 is a critical regulator of HR in *S. pombe*. However, even in this model organism, where Swi5‐Sfr1 is best characterised, the molecular role of Swi5‐Sfr1 in HR is not fully understood. Furthermore, the interplay between Swi5‐Sfr1 and other recombination accessory factors, such as the Rad51 paralogues Rad55‐Rad57, is poorly characterised at best. Do the two complexes physically interact? In what way do their stimulatory effects on Rad51/Dmc1 differ (e.g. strand invasion or strand exchange)? Related to this, can they synergistically stimulate Rad51/Dmc1? Why does Swi5‐Sfr1 have a specific regulatory mechanism mediated by Rrp1/Rrp2 30, 31? In *S. cerevisiae*, where Sae3‐Mei5 is meiosis‐specific, why is it that Rad51 does not require a Swi5‐Sfr1 homologue for mitotic HR? In addition to these questions, the possibility of post‐translational modifications regulating Swi5‐Sfr1/Sae3‐Mei5 has not been explored, although phosphorylation of both Swi5 and Sfr1 has been reported 70, 71. Collectively, the answers to these questions will allow us to begin understanding why there is a requirement for several different groups of recombination accessory proteins.

In comparison to the yeasts, relatively little is known about mammalian SWI5‐SFR1. Despite recent advances in the biochemical characterisation of mouse SWI5‐SFR1, the human complex, which is yet to be studied *in vitro*, remains an enigma. It is not known whether human SWI5‐SFR1 can exert any stimulatory effect on RAD51‐mediated strand exchange *in vitro*. Likewise, it is not known if SWI5‐SFR1 plays a role in mammalian gametogenesis. Although sequences of Swi5‐Sfr1 homologues from vertebrates and fungi have been deposited into the National Center for Biotechnology Information databases, to our knowledge, there are no known Swi5‐Sfr1 homologues in plants. If plants do not possess a Swi5‐Sfr1 homologue, it would be of interest to determine whether other recombination accessory proteins (e.g. Rad51 paralogues), or recombinases themselves, compensate for this. Future work in *S. pombe* should focus on illuminating the precise contribution of Swi5‐Sfr1 and other accessory factors to HR, which will be achieved by elucidating the various mechanisms of Rad51 stimulation. Furthermore, structural studies are required to identify the potential mechanism of RAD51 stimulation by mouse SWI5‐SFR1. Additionally, it would be of interest to determine whether mouse SWI5‐SFR1 acts synergistically with the RAD52/BRCA2 family of recombination mediators, as was reported for *S. pombe* 48. Purification of human SWI5‐SFR1 is a priority, as characterisation of its biochemical properties may reveal the extent to which the mechanism underlying eukaryotic HR has been conserved. The answer to this question may go some way to helping us appreciate the true complexity of HR defects that underlie tumorigenesis and infertility.

## Author contributions

This manuscript was prepared by BA with guidance from YM and HI.
